# Risk Factors for Recurrence of the Anti‐Synthetase Syndrome Related Interstitial Lung Disease

**DOI:** 10.1002/iid3.70417

**Published:** 2026-04-20

**Authors:** Zhen‐Yu Ren, Xue Yang, Jing Yang, Ping Kou, Xiao‐Kui Tang

**Affiliations:** ^1^ Department of Respiratory and Critical Medicine The First Affiliated Hospital of Chongqing Medical University Chongqing China; ^2^ Chongqing Red Cross Hospital Chongqing China

**Keywords:** anti‐synthetase syndrome, interstitial lung disease, recurrence

## Abstract

**Purpose:**

This current study aimed to detect the risk factors for recurrence of the anti‐synthetase syndrome related interstitial lung disease (ASSD‐ILD).

**Methods:**

Patients who were diagnosed with ASSD‐ILD and achieved the improving standards after treatment at a single clinical center from January 1, 2017 to January 1, 2022 were retrospectively collected. The patients were divided into the recurrence group and non‐recurrence group. We compared the baseline information, pulmonary function test, chest computed tomography/high‐resolution computed tomography (CT/HRCT) images, and treatments et al between the two groups. Cox regression was performed to find risk factors for recurrence. Moreover, the Kaplan‐Meier curve was conducted to estimate the recurrence‐free rate.

**Results:**

Totally 76 ASSD‐ILD patients were collected in this study. There were 24 patients in the recurrence group and 52 patients in the non‐recurrence group. The recurrence rate was 31.6%. Univariate analysis showed that pyrexia of unknown origin, neutrophil percentage > 75%, non‐specific interstitial pneumonia (NSIP) alone pattern, and immunosuppressants discontinuation were associated with recurrence (*p* < 0.05). In the primary IPTW‐weighted Cox model, pyrexia of unknown origin was significantly associated with recurrence (HR = 2.70, 95% CI: 1.12–6.51, *p* = 0.027). In sensitivity analyses using a doubly robust IPTW‐weighted Cox model, both pyrexia of unknown origin (HR = 5.17, 95% CI: 1.94–13.78, *p* = 0.001) and NSIP alone pattern (HR = 0.30, 95% CI: 0.11–0.80, *p* = 0.016) remained significantly associated with recurrence. Furthermore, the Kaplan‐Meier curve demonstrated that the non‐NSIP group (*p* = 0.039) and the pyrexia of unknown origin group (*p* = 0.023) had worse recurrence‐free rate.

**Conclusion:**

Among patients with ASSD‐ILD there is a high frequency of recurrence. The results suggested that pyrexia of unknown origin was a risk factor for recurrence. A follow‐up strategy, customized treatment, and early surveillance for high‐risk patients who relapse were critical to enhancing patients' quality of life and prognosis.

## 1 Introduction

Anti‐synthase syndrome (ASSD) was a kind of idiopathic inflammatory myopathy (IIM), which characterized by positive anti‐aminoacyl‐transfer RNA synthase(anti‐ARS) autoantibodies [1]. To date, there are 10 known antisynthetase antibodies, with anti–Jo‐1 antibody being the most common [2]. The pathogenesis of ASSD‐ILD is associated with autoimmune triggering, resulting in inflammation, pulmonary fibrosis, and remodeling of the lung parenchyma [3]. The clinical features of this disease included interstitial lung disease (ILD), arthritis, myositis, Raynaud's phenomenon, pyrexia of unknown origin, and mechanics' hand et al [4, 5, 6]. Multimodality immunosuppressants therapy is frequently necessary for anti‐synthetase syndrome patients in order to manage the disease's muscle and/or pulmonary manifestations [7].

Most patients with ASSD‐ILD had a well short‐term prognosis and a significant tendency to recurrence. The recurrence rate of ASSD‐ILD patients had been reported to be about 30.0%–56.0% [8, 9, 10]. The recurrence of ASSD‐ILD may result in acute disease progression and adversely impact patient prognosis. Therefore, it is crucial to identify the risk factors associated with ASSD‐ILD recurrence, assess the risk of recurrence, and inform treatment strategies accordingly. Moreover, there is a dearth of research on recurrence predictors in individuals with ASSD‐ILD. Previous studies have explored that both serum LDH and ground‐glass opacity (GGO) are predictors of relapse in Polymyositis (PM) or dermatomyositis (DM)‐ILD patients [11]. According to prior studies, the increase of Krebs von den Lungen‐6 (KL‐6), ferritin, surfactant protein D (SP‐D) and discontinuation of calcineurin inhibitors (CNI) were associated with the long‐term prognosis and recurrence of ASSD‐ILD [8, 10, 12].

Despite the fact that ASSD‐ILD recurrence is problematic, the knowledge of recurrence of ASSD‐ILD remains limited and controversial. Therefore, the purpose of this study was to identify the risk factors for recurrence of ASSD‐ILD and improve understanding of this condition.

## 2 Materials and Methods

### 2.1 Patients

**Figure 1:**
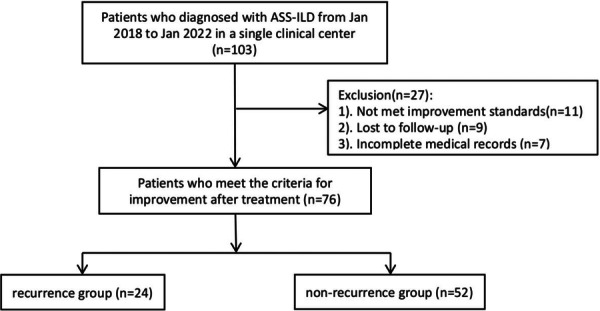
Flow chart of patients selection.

103 patients who were diagnosed with ASSD‐ILD from January 1, 2017 to January 1, 2022 at a single clinical center were retrospectively identified. This study was accordance with the Helsinki Declaration. This study has been approved by the First Affiliated Hospital of Chongqing Medical University for clinical ethics (2020‐294), and all participants signed the informed consent forms. The inclusion criteria were as follows: 1, Age ≥ 18 years old; 2, Patients who were diagnosed with ASSD‐ILD and achieved the improving standards; 3, The clinical and follow‐up data were basically complete. The exclusion criteria included: 1, Not met improvement standards (*n* = 11); 2, Lost of follow‐up (*n* = 9); and 3, Incomplete medical records (*n* = 7). In total, 76 patients were collected in this study. There were 24 patients in the recurrence group and 52 patients in the non‐recurrence group (Figure 1).

### 2.2 Data Collection

The baseline information included age, sex, smoking, and body mass index (BMI). The other information encompassed clinical feature, signs, laboratory tests, CT/HRCT image, pulmonary function, and therapeutic regimens. All the clinical data were collected from the electronic medical record system and telephone interviews.

### 2.3 Autoantibody Detection

All patients were screened for myositis antibody profiles. Myositis autoantibodies (anti‐Jo1, anti‐PL‐7, anti‐PL‐12, anti‐EJ, anti‐OJ, anti‐SRP, anti‐Mi‐2, anti MDA5, anti‐TIF1‐γ, anti NXP2, anti SAE, anti‐HMGCR, anti‐Ku, anti‐PM‐Scl75, anti‐PM‐Scl100, and anti‐Ro‐52) were measured by immunoblotting (myositis autoantibodies test kit, Shenzhen YHLO Biotech Co. Ltd., China). Test according to the manufacturer's protocol. The results were classified as positive, intermediate, and negative based on the laboratory‐recommended threshold values, which were > 10 AU/mL, 5–10 AU/mL, and < 5 AU/mL, respectively.

### 2.4 Definitions

The diagnosis of ASSD was based on the Solomon diagnostic criteria 2011 [13]. It mainly included two main criteria and 3 secondary criteria. The main criteria included 1, ILD of unknown cause; and 2, PM or DM consistent with Bohan and Peter's diagnosis [14, 15]. The secondary criteria involved 1, arthritis; 2, Raynaud's phenomenon; and 3, Mechanics' hand. On the basis of positive anti‐ARS antibodies, ASSD can be diagnosed if “2 main criteria” or “1 main + 2 secondary criteria” were met. The diagnostic criteria for ILD and the chest imaging classification criteria were based on the 2013 American Thoracic Society/European Respiratory Society (ATS/ERS) [16]. Patients who met the criteria for improvement and later met the criteria for deterioration were defined as relapsing. Improvement was defined as meeting meet two or more of the following criteria: 1, improvement of dyspnea and extra‐pulmonary symptoms associated with ASSD including myasthenia, myalgia, and rashes; 2, ILD‐associated lesions were reduced on chest CT/HRCT imaging; and 3, The percentage of predicted forced vital capacity (FVC%) increased by ≥ 10%, the percentage of diffusing capacity of the lung carbon monoxide (D_L_CO%) increased by ≥ 15%, the arterial oxygen tension (PaO_2_) increased by ≥ 10 mmHg, or the peripheral oxygen saturation (SpO_2_) increased by ≥ 4%. The deterioration criteria needed to meet two or more of the following criteria: 1, Exacerbation of dyspnea or extra‐pulmonary symptoms associated with ASSD; 2, ILD‐associated lesions increased on chest CT/HRCT imaging; 3, FVC% reduced by ≥ 10%, D_L_CO% reduced by ≥ 15%, PaO_2_ reduced by ≥ 10 mmHg, or SpO_2_ reduced by ≥ 4%; and 4, new developments in ASSD‐related extrapulmonary manifestations [17, 18, 19]. Disagreement would be solved by our multidisciplinary team including department of respiratory, rheumatology, and radiologist.

### 2.5 Statistics

GPower 3.1 was used to perform calculations on sample size. The minimal significance (α) and statistical power (1 − *β*) were set at 0.05 and 0.80 respectively. The ratio of sample size between the two groups was 0.3. The total sample size was 74. Our final sample size was 76 patients, with 52 patients in the non‐recurrence group and 24 patients in the recurrence group. Missing values are processed using multiple imputation techniques. Continuous variables with a normal distribution are described as mean ± standard deviation (SD), continuous non‐normal variables are described as median and interquartile range (IQR), and categorical variables are described as absolute and relative frequencies. Independent sample *t*‐test was used to compare the difference for continuous variables with a normal distribution. Mann‐Whitney U test was used for the data with non‐normal distribution. Chi‐square test and Fisher's exact test were adopted for categorical variables. *p* < 0.05 were set as statistically significant. The Bonferroni correction was used when necessary. Multicollinearity was checked using VIF. The Cox proportional hazards assumption was checked using the Schoenfeld residual test, and the results were consistent with the assumption. Multivariate Cox regression analysis was conducted to identify risk factors for recurrence. To further validate the robustness of our findings, inverse probability of treatment weighting (IPTW) based on propensity scores was applied. Stabilized IPTW weights were incorporated into Cox proportional hazards models to estimate the marginal association between exposure and relapse. The primary IPTW‐weighted Cox model included exposure only, estimating the population‐average effect. In addition, a doubly robust IPTW‐weighted Cox model was fitted by further adjusting for baseline covariates (age, sex, and radiologic pattern) as a sensitivity analysis. Robust sandwich variance estimators were used in all weighted models. The Kaplan‐Meier method was used to assess the recurrence‐free rate between the different group. Data were analyzed using SPSS (version 26.0) software, and a two‐tailed *p* < 0.05 was deemed as significant difference. Propensity scores were estimated using logistic regression in SPSS (version 26.0). Inverse probability of treatment weights (IPTW) were calculated based on the estimated propensity scores. Because SPSS does not support Cox proportional hazards models with continuous weights, weighted Cox regression analyses were conducted in R (version 4.4.2) using the survival package. The same dataset and IPTW values were used consistently across all analyses.

## 3 Results

### 3.1 Baseline Characteristics

In this study, a total of 76 ASSD‐ILD patients were collected. There were 24 participants in the recurrence group and 52 patients in the non‐recurrence group. There were 58 females in the total cohort. The age at diagnosis ranged from 18 to 75 years, with an average age of 57.2 ± 10.8 years. The median follow‐up time was 38.0 (IQR: 22.8) months. No significant difference was found in the baseline characteristics between the two groups.

### 3.2 Clinical Manifestation

According to Solomon's ASSD diagnostic criteria, a total of 40 participants (52.6%) met the “2 main criteria” and 36 participants (47.4%) were diagnosed with ASSD through “1 main criterion+2 secondary criteria”. There was a total of 16 participants (21.1%) with myositis, arthritis, and ILD triad. Approximately one‐third (34.2%) manifested as acute/subacute presentations within a timeframe of 1–3 months, whereas the majority (65.79%) demonstrated a chronic/insidious progression lasting beyond 3 months or even in the absence of apparent respiratory symptoms [18]. The clinical manifestations mainly include respiratory symptoms (85.5%), rash (25.0%), arthritis (53.9%), mechanics' hand (44.7%), Raynaud's phenomenon (34.2%), and pyrexia of unknown origin (13.2%). More details about clinical manifestation of the total cohort were shown in the Table 1.

**Table 1:** Univariate analysis of risk factors for ASSD‐ILD.

Clinical characteristics	Overall (*n* = 76)	Recurrence group (*n* = 24)	Non‐recurrence group (*n* = 52)	*p* value
Female	58 (76.3)	20 (83.3)	38 (73.1)	0.328
Age ≥ 65 years	20 (26.3)	9 (37.5)	11 (21.2)	0.133
BMI, kg/m^2 b^	23.6 ± 3.2	23.7 ± 2.5	23.5 ± 3.5	0.728
Smoking history	16 (21.1)	3 (12.5)	13 (25.0)	0.214
Primary symptom				0.408
Respiratory symptom	45 (59.2)	12 (50.0)	33 (63.5)	
Non‐respiratory symptom	23 (30.3)	8 (33.3)	15 (28.8)	
Meantime	8 (10.5)	4 (16.7)	4 (7.7)	
Onset of ILD (Acute/Subacute)	27 (35.5)	11 (45.8)	16 (30.8)	0.202
Respiratory symptom (entire follow‐up period)^a^	65 (85.5)	21 (87.5)	44 (84.6)	1.000
Rash	19 (25.0)	3 (12.5)	16 (30.8)	0.087*
Arthritis	41 (53.9)	13 (54.2)	28 (53.8)	0.979
Mechanics' hand	34 (44.7)	11 (45.8)	23 (44.2)	0.896
Raynaud's phenomenon	26 (34.2)	7 (29.2)	19 (36.5)	0.529
Pyrexia of unknown origin	10 (13.2)	7 (29.2)	3 (5.8)	0.015*
Crackle	36 (47.4)	12 (50.0)	24 (46.2)	0.755
Respiratory failure	10 (13.2)	4 (16.7)	6 (11.5)	0.803
Myositis + arthritis + ILD	16 (21.1)	5 (20.8)	11 (21.2)	0.975
Diagnostic criteria (two main criteria)	40 (52.6)	14 (58.3)	26 (50.0)	0.499
Type of anti‐ARS antibodies				0.726
Anti‐Jo‐1 antibody	47 (61.8)	13 (54.2)	34 (65.4)	0.349
Anti‐PL‐7 antibody	8 (10.5)	3 (12.5)	5 (9.6)	1.000
Anti‐PL‐12 antibody	8 (10.5)	4 (16.7)	4 (7.7)	0.434
Anti‐EJ antibody	12 (15.8)	4 (16.7)	8 (15.4)	1.000
Anti‐OJ antibody	1 (1.3)	0 (0)	1 (1.9)	1.000
Positive anti‐Ro‐52 antibody	53 (69.7)	19 (79.2)	34 (65.4)	0.224
Positive ANA with high titer	26 (34.2)	9 (37.5)	17 (32.7)	0.681
WBC > 9.5*10^9/L	23 (30.3)	8 (33.3)	15 (28.8)	0.692
Hb < 130 g/L	36 (47.4)	13 (54.2)	23 (44.2)	0.420
Lymphocyte% < 20	43 (56.6)	15 (62.5)	28 (53.8)	0.479
Neutrophil% > 75	32 (42.1)	6 (25.0)	26 (50.0)	0.040*
Elevated ESR (mm/h)	60 (78.9)	20 (83.3)	40 (76.9)	0.524
Decreased serum level of C3 or C4^b^	28 (36.8)	8 (33.3)	20 (38.5)	0.667
Elevated muscle enzymes (LDH/ALT/AST/CK)	42 (55.3)	16 (66.7)	26 (50.0)	0.174
Pulmonary function test				
FVC%^b^	84.0 (31.2)	84.0 (30.8)	85.0 (32)	0.801
FEV1%^b^	80.0 (23.5)	80.0 (25.3)	79.5 (24.5)	0.951
D_L_CO%^b^	57.0 (36.3)	52.0 (32.8)	67.3 (39.2)	0.647
CT/HRCT pattern of ILD				0.032*
NSIP alone	51 (67.1)	12 (50.0)	39 (75.0)	0.031*
OP	8 (10.5)	5 (20.8)	3 (5.8)	0.113
NSIP with OP	14 (18.4)	7 (29.2)	7 (13.5)	0.186
UIP^a^	3 (3.9)	0 (0)	3 (5.8)	0.547
Treatment				
Immunosuppressants	68 (89.5)	22 (91.7)	46 (88.5)	0.510
CTX	43 (56.6)	11 (45.8)	32 (61.5)	0.199
CsA	9 (11.8)	5 (20.8)	4 (7.7)	0.205
MMF	9 (11.8)	4 (16.7)	5 (9.6)	0.615
MTX	3 (3.9)	1 (4.2)	2 (3.8)	1.000
AZA	4 (5.3)	1 (4.2)	3 (45.8)	1.000
Immunosuppressants discontinuation^b^	36 (52.2)	16 (69.6)	20 (43.5)	0.041*
Immunoglobin	9 (11.8)	4 (16.7)	5 (9.6)	0.615
Mechanical ventilation^a^	5 (6.6)	2 (8.3)	3 (5.8)	0.648

*Note:* ^a^Fisher's exact test, ^b^there are missing values, Variables are expressed as the mean ± SD, *n* (%), median (range), Mean or median values and proportions were compared between two groups using the Mann‐Whitney *U* test, Student's t test, or chi‐square test, *p < 0.1. Positive ANA with a high titer: the titer of nucleolar or centromeric pattern ≥ 1:100 or the titer of non‐nucleolar or centromeric pattern ≥ 1:320. Elevated ESR, ESR > 43 mm/h (man, age > 60 years) or > 21 mm/h (man, age ≤ 60 years) or > 38 mm/h (woman aged > 50 years) or > 26 mm/h (woman aged ≤ 50 years). Decreased serum level of C3 or C4, serum C3 < 0.79 g/L or serum C4 < 0.16 g/L. Elevated muscle enzymes: lactate dehydrogenase > 250 U/L or aspartate aminotransferase > 35 U/L or alanine aminotransferase > 35 U/L or creatine kinase > 200 U/L.

Abbreviations: ALT, alanine aminotransferase; ANA, anti‐nuclear antibody; anti‐ARS antibodies, anti‐aminoacyl‐transferase RNA synthetase (ARS) antibodies; AST, aspartate transaminase; AZA, azathioprine; BMI, body mass index; C3 or C4, Complement 3 or Complement 4; CK, creatine kinase; CsA, cyclosporine; CT/HRCT, computed tomography/high‐resolution computed tomography; CTX, cyclophosphamide; DLCO%, the percentage of diffusing capacity of the lung carbon monoxide; ESR, erythrocyte sedimentation rate; FEV1%, percentage of forced expiratory volume in 1 s; FVC%, the percentage of predicted forced vital capacity; ILD, interstitial lung disease; LDH, lactate dehydrogenase; MMF, mycophenolate mofetil; MTX, methotrexate; NSIP, nonspecific interstitial pneumonia; OP, organizing pneumonia; UIP, usual interstitial pneumonia; WBC, white blood cell, Hb, hemoglobin.

### 3.3 Laboratory Tests

Among the 76 participants, 47 (61.8%) were anti‐Jo‐1 positive, 8 (10.5%) were anti‐PL‐7 positive, 8 (10.5%) were anti‐PL‐12 positive, 12 (15.8%) were anti‐EJ positive and 1 (1.3%) were anti‐OJ positive. 26 participants (34.2%) were positive for anti‐nuclear antibody (ANA; high titer) and 53 (69.7%) were positive for the anti‐Ro‐52 antibody. The white blood cell (WBC) count and neutrophil% were increased in 23 participants (30.3%) and 32 participants (42.1%), respectively. The hemoglobin (Hb) and lymphocyte% were decreased in 36 participants (47.4%) and 43 participants (56.6%), respectively. Erythrocyte sedimentation rate (ESR) was increased in 60 participants (78.9%) and serum level of C3 or C4 decreased in 28 participants (36.8%). The muscle enzymes were increased in 42 (55.3%) participants.

### 3.4 Pulmonary Function and Chest CT/HRCT Pattern of LID

**Figure 2:**
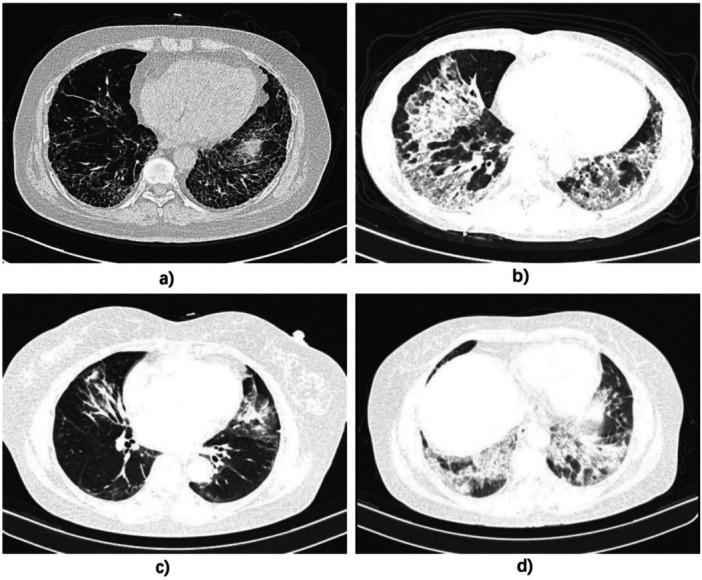
Typical HRCT images for ASS‐ILD patients. (a) HRCT image in an anti‐EJ antibody‐positive ASS‐ILD patient, which exhibited UIP pattern and the patient experienced recurrence at 18 months after diagnosis. (b) HRCT image in an anti‐Jo‐1 antibody‐positive ASS‐ILD patient, which exhibited NSIP with OP pattern and the patient has not experienced recurrence. (c) HRCT image in an anti‐Jo‐1 antibody‐positive ASS‐ILD patient, which exhibited NSIP pattern and the patient experienced recurrence at 8 months after diagnosis. (d) HRCT image in an anti‐Jo‐1 antibody‐positive ASS‐ILD patient, which exhibited OP pattern and the patient has not experienced recurrence.

A total of 56 patients (73.7%) completed pulmonary function test, median FVC% was 84.0 (IQR: 31.2), median percentage of forced expiratory volume in 1 s (FEV1%) was 80.0 (IQR: 23.5), and median D_L_CO% was 57.0 (IQR: 36.3). Chest CT/HRCT images were available for all patients. NSIP alone pattern was observed in 51 participants (67.1%), OP pattern was observed in 8 participants (10.5%), NSIP with OP pattern was observed in 14 participants (18.4%) and UIP pattern was observed in 3 participants (3.9%). Figure 2 shows some typical CT/HRCT images of ASSD‐ILD patients.

### 3.5 Treatments

All participants were treated with glucocorticoid, with 8 participants (10.5%) choosing glucocorticoid as monotherapy and 68 participants (89.5%) choosing glucocorticoid combined with immunosuppressants. 43 participants (56.6%) received cyclophosphamide (CYC) treatment, 9 participants (11.8%) received cyclosporine (CsA), 9 participants (11.8%) received mycophenolate mofetil (MMF), 3 participants (3.9%) received methotrexate (MTX), 4 participants (5.3%) received azathioprine (AZA). A total of 9 patients (11.8%) received immunoglobulin therapy and 5 patients (6.6%) received mechanical ventilation, including 2 cases of non‐invasive mechanical ventilation and 3 cases of invasive mechanical ventilation.

### 3.6 Clinical Characteristics of Recurrence Patients

During the follow‐up period, 24 participants (31.6%) experienced recurrence with the median period of 28.0 (IQR: 24.0) months until the first recurrence. A total of 10 participants died, with an all‐cause mortality rate of 13.2%. Two participants died of ASSD‐ILD during the first recurrence, and the remaining eight patients died of other causes. Among the 68 participants receiving glucocorticoid combined with immunosuppressants therapy, a total of 23 cases (33.8%) experienced recurrence. 9 participants (39.1%) reported that the recurrence was secondary to pulmonary infection. Before the recurrence, 15 (65.2%) participants had already stopped using immunosuppressants and 7 participants (30.4%) discontinued glucocorticoid. The average dose of glucocorticoid of 23 relapsed participants at the time of recurrence was 7.4 ± 6.9 mg/day, of which 7 participants (73.9%) had a glucocorticoid dose ≤ 10 mg/day. All patients were retreated or adjusted the immunosuppressants after recurrence (10 with CYC, 3 with CsA, 5 with MMF, 2 with tacrolimus, 3 with AZA). More than 78% of participants have effectively controlled their condition once more.

### 3.7 Univariate and Multivariate Analysis of Recurrence

We performed a univariate analysis on the demographic characteristics, clinical manifestations, laboratory tests, CT/HRCT pattern, pulmonary function, and therapeutic regimens of 76 patients (Table 1 and Table S1). In the univariate analysis, we found that the recurrence group patients had a more portion of pyrexia of unknown origin (*p* = 0.015), less neutrophil% > 75 (*p* = 0.040), less NSIP alone pattern (*p* = 0.031), and more immunosuppressants discontinuation (*p* = 0.041). CT/HRCT pattern of ILD showed an association (*p* = 0.032) with recurrence, and there was no significant difference between them after pairwise comparison with Bonferroni correction. In addition, a subgroup analysis within patients with NSIP suggested that those with overlapping OP tended to have a higher risk of recurrence compared with NSIP alone (HR = 2.507, 95% CI= 0.970–6.478; *p* = 0.058) (Table S3), although this association did not reach statistical significance, likely due to the limited sample size. In IPTW‐weighted Cox regression analyses, pyrexia of unknown origin was significantly associated with an increased risk of relapse (HR: 2.70, 95% CI: 1.12–6.51, *p* = 0.027). In the doubly robust IPTW‐weighted Cox model additionally adjusting for age, sex, and radiologic pattern, the association between pyrexia of unknown origin and relapse remained statistically significant (HR 5.17, 95% CI: 1.94–13.78, *p* = 0.001)（Table 2). In this model, NSIP pattern was also associated with a lower risk of relapse (HR: 0.30, 95% CI: 0.11–0.80, *p* = 0.016) (Table S2).

**Table 2:** IPTW‐weighted Cox proportional hazards models for relapse.

Model	Analysis type	Variables included	HR (95% CI)	*p* value
Model 1 (Primary)	IPTW‐weighted Cox	Pyrexia of unknown origin	2.70 (1.12–6.51)	0.027*
Model 2 (Sensitivity)	Doubly robust IPTW Cox	Pyrexia of unknown origin + age + sex + NSIP	5.17 (1.94–13.78)	0.001*

*Note:* Model 1 estimates the marginal association between fever of unknown origin and relapse using an IPTW‐weighted Cox model. *p < 0.05. Model 2 is a doubly robust IPTW‐weighted Cox model additionally adjusting for age, sex, and radiologic pattern.

Abbreviations: IPTW, inverse probability of treatment weighting; NSIP, non‐specific interstitial pneumonia.

### 3.8 Kaplan‐Meier Curves

**Figure 3:**
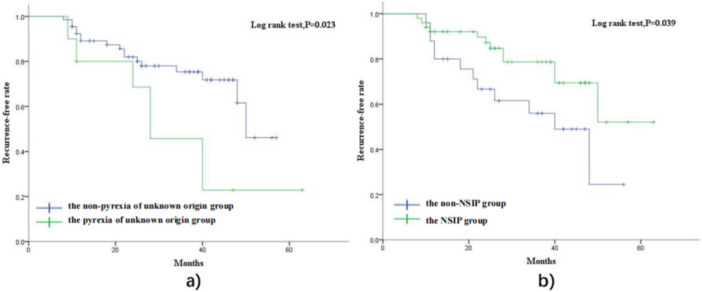
Estimated recurrence‐free rate by Kaplan‐Meier method. (a) The pyrexia of unknown origin group and non‐pyrexia of unknown origin. (b) The NSIP group and non‐NSIP group.

The ASSD‐ILD recurrence‐free rates in participants analyzed by the Kaplan‐Mayer method. As shown in Figure 3, the recurrence‐free rate of participants with pyrexia of unknown origin is significantly lower than that of participants without pyrexia of unknown origin (*p* = 0.023). And the recurrence‐free rate of participants with NSIP pattern is significantly higher than that of participants with non‐NSIP pattern (*p* = 0.039).

## 4 Discussion

This current study analyzed the clinical features, treatment content and recurrence of ASSD‐ILD in 76 patients diagnosed in the First Affiliated Hospital of Chongqing Medical University from January 1, 2017 to January 1, 2022. In this study, the recurrence rate of ASSD‐ILD patients was 31.6%, which was consistent with previous studies. The univariate analysis indicated that pyrexia of unknown origin, neutrophil% > 75%, NSIP alone pattern, and immunosuppressants discontinuation were associated with recurrence. After accounting for baseline imbalance using IPTW, pyrexia of unknown origin showed a robust association with recurrence. This association remained consistent in doubly robust analyses, in which NSIP pattern was additionally associated with a lower risk of recurrence.

ASSD was more common in women than in men. The age of onset in adults ranged from 19 to 82 years, with an average age of 43–60 years, which was familiar with our study [2, 20]. ASSD usually involved varieties of organs such as lungs, muscles, joints, and skin, among which the overall prevalence of ILD in ASSD patients is about 70%–95% [7, 21, 22, 23]. ASSD‐ILD had a tendency to relapse, and the recurrence rate varies in different study cohorts, ranging from 30.0% to 56.0% [8, 9, 10], which was consistent with our study. A retrospective study showed that anti‐ARS antibodies positivity was the risk factors for the recurrence of ILD with PM/DM [24]. Isoda et al thought that the recurrence rate of PM/DM patients with positive anti‐ARS antibodies was higher than that of patients with positive anti‐melanoma differentiation‐related genes (MDA5), and the ferritin and KL‐6 levels in the former are significantly higher than those in the latter [10]. This indicated that compared to other IIM, ASSD‐ILD was more likely to reoccur. On the other hand, Takei et al demonstrated that the increase of KL‐6 and the discontinuation of CNI might lead to recurrence [8]. A study suggested that pyrexia, lower counts of CD3 + CD4+ cells, and UIP pattern were independent risk factors of ASSD‐ILD [25]. The recurrence of ASSD‐ILD seriously affected the quality of life and prognosis of patients, so it is of great clinical significance to search for the risk factors of ASSD‐ILD recurrence. At present, there were few studies focus on the risk factors for recurrence of ASSD‐ILD. Therefore, we analyzed and discussed the risk factors of recurrence in ASSD‐ILD patients, in order to help for the identification of patients at high risk for recurrence and the formulation of treatment strategies.

In this study, the independent risk factors for recurrence were pyrexia of unknown origin. There were 10 patients with pyrexia of unknown origin, with 7 cases (29.2%) in the recurrence group and 3 cases (5.8%) in the non‐recurrence group. In the study of Ahn et al, pyrexia was identified as an independent risk factor for mortality in patients with PM/DM [26]. As previously study showed pyrexia is a risk factor associated with the disease's progression in patients with ASSD‐ILD [25]. Patients with ASSD‐ILD who presented with recurrent episodes of fever and elevated acute phase reactants due to systemic inflammation were deemed to be more aggressive and more refractory to conventional immunosuppressants therapies [27]. This is supported by previous studies demonstrating increased expression of pyrogenic cytokines such as tumor necrosis factor‐α(TNF‐α), interleukin‐1(IL‐1), interleukin‐6(IL‐6) and interferon‐γ(IFN‐γ) in the circulation and muscles in patients with IIM [28, 29, 30]. One study suggested that neutrophil‐to‐lymphocyte ratio, C‐reactive protein‐to‐albumin ratio, systemic inflammatory index, and systemic inflammatory response index may represent disease activity in ASSD patients [3]. Liu et al. [31] showed that the significantly higher levels of IL‐6 in patients with ASSD may be associated with disease progression and recurrence. Our study is in agreement with previous clinical that a higher degree of systemic inflammatory response is suggested by pyrexia in patients with ASSD‐ILD. These individuals are more prone to recur during long‐term maintenance treatment because of their worse sensitivity to immunosuppressants and glucocorticoid.

The most common CT/HRCT image pattern is NSIP, which was similar to our research findings [32, 33, 34, 35]. In sensitivity analyses using a doubly robust IPTW‐weighted Cox model, NSIP pattern was associated with a reduced risk of recurrence. The NSIP pattern characterized by patchy or diffuse ground‐glass opacities (GGO) with associated reticular opacities, and UIP pattern characterized by bilateral subpleural reticulation and honeycombing with minimal or nonexistent GGO. According to Liu et al's research, NSIP and NSIP‐OP are associated to a better prognosis in ASSD‐ILD patients than UIP [25]. In the case of Marie et al's research, there is a strong link between UIP and ILD deterioration [36]. A retrospective study reported that ASSD‐ILD patients with disease progression showed an increase or new development of honeycombing [34]. Overall, previous research has demonstrated that in various imaging manifestations of ASSD‐ILD patients, NSIP is considered to have a better prognosis than UIP. UIP is the advancement of pulmonary fibrosis in patients for whom hormones and immunosuppressants have limited effects on improving respiratory physiological function and status. This may help to explain why UIP is frequently associated with disease progression and recurrence. In our subgroup analysis, patients with NSIP overlapping OP tended to have a higher risk of recurrence compared with NSIP alone, although this association did not reach statistical significance, likely due to limited sample size. This observation suggests that the NSIP with OP may represent a distinct clinical subgroup warranting future investigation.

In our univariate analysis, there was a significant correlation between discontinuation of immunosuppressants (excluding glucocorticoid) and recurrence (*p* = 0.041). No differences were observed in the multivariate analysis, which may be related that our sample was relatively small. For ASSD‐ILD patients, immunosuppressants such as CNI, MTX, AZA, MMF, and CYC help alleviate clinical symptoms and lower recurrence rates [24, 37, 38, 39]. CNI have received more attention in this regard. A recent study reported that CNI discontinuation was significantly associated with the recurrence of ASSD‐ILD. In this study, the CNI continued and CNI discontinued groups in this research had recurrence rates of 39% and 89%, accordingly [8]. In the study of Hozumi et al., ASSD‐ILD patients were treated with glucocorticoid or glucocorticoid +CNI respectively, and the latter group had significantly higher 2‐year progression‐free survival and lower recurrence rates [18]. In the expert consensus, it is also pointed out that glucocorticoid monotherapy has a higher recurrence rate in patients with ASSD‐ILD. The first‐line treatment of ASSD ILD is advised to combine glucocorticoid and immunosuppressants [40]. In the initial therapeutic process, the determination of which immunosuppressive agents to select, especially for patients with a high propensity for recurrence is of paramount importance. Moreover, there is a pressing need for relevant research to provide guidance on the modulation of immunosuppressive agents after relapse to inform clinical practice. Larger, independent, multicenter studies are mandatory to thoroughly evaluate treatment for immunosuppressants, including the type of medication, duration of use, and maintenance dose for ASSD‐ILD.

73.9% of participants in our research who were in the recurrence group had glucocorticoid ≤ 10 mg/d. In previous studies, the reduction of glucocorticoid is associated with the recurrence of ASSD‐ILD [24, 41]. Chen et al. discovered that GC tapering duration and GC discontinuation were independent predictors for recurrence of ASSD‐ILD [19]. Due to limitations in retrospective studies, we were unable to further analyze the relationship between glucocorticoid dosage and recurrence. Anticipating pertinent studies that will inform clinical strategies for glucocorticoid reduction in the future.

The study has several limitations. First, this study was a single‐center retrospective study, and some examination data were missing. For example, pulmonary function tests are not completed in all patients. Previous studies suggesting worse pulmonary function predicts unfavorable outcome in ASSD‐ILD patients [24, 42]. However, no significant difference in pulmonary function was observed between the recurrence and non‐recurrence groups in our study, and larger studies may be needed to analyze the effect of pulmonary function on recurrence rates. Second, the follow‐up time of the 76 patients varied, and there was insufficient analysis of long‐term outcomes and death.

In this study, the recurrence rate of ASSD‐ILD patients was 31.6%. Patients who had a pyrexia of unknown origin were more likely to experience recurrences. This gives us some suggestions when designing individualized therapy and follow‐up schedules for patients with ASSD‐ILD. For patients with ASSD‐ILD, it is imperative to enhance the initial treatment and follow‐up procedures for those had pyrexia of unknown origin. Additionally, a reinforced follow‐up and continuous treatment are also crucial, aiming to mitigate recurrence risk, enhance prognosis, and improve overall patient quality of life. Considering the high risk of recurrence, it is recommended to initiate treatment with a combination of glucocorticoid and immunosuppressant therapy. In the selection of immunosuppressants, CNI can be given priority, while caution should be exercised when discontinuing immunosuppressants. Our findings have the potential to inform the design of future studies aimed at determining the optimal treatment strategy for patients with ASSD‐ILD.

## 5 Conclusion

Disease recurrence significantly influenced the long‐term prognosis of ASSD‐ILD. This retrospective study is valuable that it offers a foundation for early detection of patients who are at high risk of relapsing. Further prospective and randomized controlled studies are warranted to elucidate risk factors for ASSD‐ILD and to devise optimal treatment strategies.

## Author Contributions

**Zhen‐Yu Ren:** data curation, formal analysis, visualization, writing – original draft, writing – review and editing. **Xue Yang:** data curation, investigation. **Jing Yang:** data curation, investigation. **Ping Kou:** data curation, investigation. **Xiao‐Kui Tang:** conceptualization, methodology, project administration, supervision, validation.

## Funding

The authors have nothing to report.

## Ethics Statement

This study has been approved by the First Affiliated Hospital of Chongqing Medical University for clinical ethics (2020–294), and the requirement for informed consent was waived due to the retrospective nature of the study.

## Consent

All patients signed the informed consent to participate in this study.

## Conflicts of Interest

The authors declare no conflicts of interest.

## Supporting information

STROBE‐checklist.

**Table S1:** Baseline characteristics before and after IPTW.

**Table S2:** Doubly robust IPTW‐weighted Cox proportional hazards model for relapse.

**Table S3:** Result of Univariate Cox proportional hazards regression.

## Data Availability

Availability of data and materials: The data are available from the corresponding author on reasonable request.
