# Supplementary material for: Risk Factors for Recurrence of the Anti‐Synthetase Syndrome Related Interstitial Lung Disease
Source: Immun Inflamm Dis. 2026 Apr 20;14(4):e70417. doi: 10.1002/iid3.70417 (PMC13096720; doi:10.1002/iid3.70417)
Supplement: Supplementary file 2 — Table S1: Baseline characteristics before and after IPTW. [file IID3-14-e70417-s001.docx]

| **Table S1. Baseline characteristics before and after IPTW** | | | | |
| --- | --- | --- | --- | --- |
| Variable | Unweighted No pyrexia (n=66) | Unweighted Pyrexia (n=10) | Weighted No pyrexia(%) | Weighted pyrexia(%) |
| Age≥65 years, n (%) | 17(25.8) | 3(30.0) | 25.8 | 25.0 |
| Female sex, n (%) | 50(75.8) | 8(80.0) | 75.8 | 62.5 |
| Smoking history, n (%) | 14(21.2) | 2(20.0) | 21.2 | 37.5 |
| Onset of ILD(Acute/Subacute), n (%) | 42(63.6) | 7(70.0) | 63.6 | 62.5 |
| Respiratory failure, n (%) | 8(12.1) | 2(20.0) | 13.6 | 12.5 |
| Anti-Jo-1 positive, n (%) | 43(65.2) | 4(40.0) | 63.6 | 25.0 |
| Anti-Ro-52 positive, n (%) | 45(68.2) | 8(80.0) | 69.7 | 75.0 |
| Neutrophil % >75%, n (%) | 28(42.4) | 4(40.0) | 42.4 | 37.5 |
| Elevated ESR, n (%) | 50(75.8) | 10(100.0) | 78.8 | 100.0 |
| Note: Values are presented as number (percentage). Weighted values represent IPTW-adjusted distributions. Unweighted values are presented as number (percentage). Weighted values represent IPTW-adjusted distributions. IPTW was used as a sensitivity analysis to assess the robustness of the primary findings. Residual imbalance in some covariates may remain due to the limited sample size.  Abbreviations: IPTW, inverse probability of treatment weighting; ILD, interstitial lung disease; ESR, erythrocyte sedimentation rate. | | | | |
