# Supplementary material for: Risk Factors for Recurrence of the Anti‐Synthetase Syndrome Related Interstitial Lung Disease
Source: Immun Inflamm Dis. 2026 Apr 20;14(4):e70417. doi: 10.1002/iid3.70417 (PMC13096720; doi:10.1002/iid3.70417)
Supplement: Supplementary file 3 — Table S2: Doubly robust IPTW‐weighted Cox proportional hazards model for relapse. [file IID3-14-e70417-s004.docx]

| **Table S2. Doubly robust IPTW-weighted Cox proportional hazards model for relapse** | | | |
| --- | --- | --- | --- |
| Variable | HR | 95% CI | P value |
| Pyrexia of unknown origin | **5.17** | **1.94–13.78** | **0.001**^*^ |
| Age | 1.87 | 0.83–4.19 | 0.132 |
| Sex | 0.78 | 0.30–2.01 | 0.605 |
| NSIP alone | **0.30** | **0.11–0.80** | **0.016**^*^ |
| Note: The doubly robust model incorporates IPTW weighting and additional adjustment for baseline covariates (age, sex, and NSIP). Robust sandwich variance estimators were applied.^*^P-value<0.05.  Abbreviations: IPTW, inverse probability of treatment weighting;NSIP, non-specific interstitial pneumonia. | | | |
