# Supplementary material for: Risk Factors for Recurrence of the Anti‐Synthetase Syndrome Related Interstitial Lung Disease
Source: Immun Inflamm Dis. 2026 Apr 20;14(4):e70417. doi: 10.1002/iid3.70417 (PMC13096720; doi:10.1002/iid3.70417)
Supplement: Supplementary file 4 — Table S3: Result of Univariate Cox proportional hazards regression. [file IID3-14-e70417-s002.docx]

| **Table S3. Result of Univariate Cox proportional hazards regression** | | | | | |
| --- | --- | --- | --- | --- | --- |
|  | β | Wald | P value | HR | 95% CI |
| NSIP with OP | 0.92 | 3.60 | 0.058 | 2.51 | 0.97-6.48 |
| Abbreviations: NSIP, non-specific interstitial pneumonia;OP, organizing pneumonia. | | | | | |
